# Automatic segmentation of hemorrhagic transformation on follow-up non-contrast CT after acute ischemic stroke

**DOI:** 10.3389/fninf.2024.1382630

**Published:** 2024-04-16

**Authors:** Jiacheng Sun, Freda Werdiger, Christopher Blair, Chushuang Chen, Qing Yang, Andrew Bivard, Longting Lin, Mark Parsons

**Affiliations:** ^1^Sydney Brain Centre, The Ingham Institute for Applied Medical Research, Liverpool, NSW, Australia; ^2^South Western Sydney Clinical School, University of New South Wales, Sydney, NSW, Australia; ^3^Melbourne Brain Centre at Royal Melbourne Hospital, Melbourne, VIC, Australia; ^4^Department of Medicine, University of Melbourne, Melbourne, VIC, Australia; ^5^Department of Neurology and Neurophysiology, Liverpool Hospital, Sydney, NSW, Australia; ^6^Apollo Medical Imaging Technology Pty. Ltd., Melbourne, VIC, Australia

**Keywords:** acute ischemic stroke, hemorrhagic transformation, endovascular thrombectomy, image segmentation, volume quantification, non-contrast CT

## Abstract

**Background:**

Hemorrhagic transformation (HT) following reperfusion therapies is a serious complication for patients with acute ischemic stroke. Segmentation and quantification of hemorrhage provides critical insights into patients’ condition and aids in prognosis. This study aims to automatically segment hemorrhagic regions on follow-up non-contrast head CT (NCCT) for stroke patients treated with endovascular thrombectomy (EVT).

**Methods:**

Patient data were collected from 10 stroke centers across two countries. We propose a semi-automated approach with adaptive thresholding methods, eliminating the need for extensive training data and reducing computational demands. We used Dice Similarity Coefficient (DSC) and Lin’s Concordance Correlation Coefficient (Lin’s CCC) to evaluate the performance of the algorithm.

**Results:**

A total of 51 patients were included, with 28 Type 2 hemorrhagic infarction (HI2) cases and 23 parenchymal hematoma (PH) cases. The algorithm achieved a mean DSC of 0.66 ± 0.17. Notably, performance was superior for PH cases (mean DSC of 0.73 ± 0.14) compared to HI2 cases (mean DSC of 0.61 ± 0.18). Lin’s CCC was 0.88 (95% CI 0.79–0.93), indicating a strong agreement between the algorithm’s results and the ground truth. In addition, the algorithm demonstrated excellent processing time, with an average of 2.7 s for each patient case.

**Conclusion:**

To our knowledge, this is the first study to perform automated segmentation of post-treatment hemorrhage for acute stroke patients and evaluate the performance based on the radiological severity of HT. This rapid and effective tool has the potential to assist with predicting prognosis in stroke patients with HT after EVT.

## 1 Introduction

For patients with acute ischemic stroke, hemorrhagic transformation (HT) is a feared complication and a leading cause of mortality following reperfusion therapies. Computed Tomography (CT) imaging remains the principal modality to diagnose post-reperfusion treatment hemorrhage (Siddiqui et al., 2011; Heit et al., 2017). Prompt detection and accurate characterization of acute hemorrhage is crucial for ongoing treatment and prognosis.

Hemorrhage volume is strongly associated with functional outcomes (de Havenon et al., 2020; van Kranendonk et al., 2020). An automated tool for quantifying HT volumes can be applied to clinical trials involving thrombolytic agents and post-treatment blood pressure modulation, where HT is a major safety outcome. It can provide an objective assessment of outcome and improve audit quality. In contrast, manual estimation tends to be subjective and biased, and requires considerable skills and experience. While the qualitative ABC/2 method and RAPID ICH (iSchemaView) are often applied clinically for detecting and quantifying primary intracerebral hemorrhage (ICH) (Kothari et al., 1996; Divani et al., 2011; Chinda et al., 2018; Heit et al., 2021; Eldaya et al., 2022), no reliable volumetric measure is available for HT.

Two main categories of methods are applied in recent studies to develop automated tools, although most of them focus on primary ICH rather than HT. The first are traditional segmentation techniques such as Fuzzy C-Means (FCM) clustering (Pham and Prince, 1999) and region-based active contour method (Caselles et al., 1993). In this category, Bhadauria et al. (2013) presented an integrated method for hemorrhage segmentation from CT of only 20 patients. Sun et al. (2015) proposed a novel three-dimensional (3D) method for segmenting hemorrhage regions from CT images, again from a small number of 20 patients. Pszczolkowski et al. (2019) introduced an automated unsupervised algorithm for hematoma segmentation from magnetic resonance (MR) images following spontaneous intracerebral hemorrhage of 50 patients. Kumar et al. (2022) developed an entropy-based method for intracranial hemorrhage segmentation using CT in 35 patients.

The second category relates to Deep Learning-based algorithms. Convolutional neural networks (CNNs) have been shown to be particularly efficient at learning image segmentation tasks. Kuo et al. (2019) trained a fully convolutional neural network (FCN) with 4,396 CT images for segmenting acute intracranial hemorrhage. Abramova et al. (2021) proposed a patch-based method for segmenting hemorrhagic stroke with 76 patient CT datasets using a modified U-Net (a subcategory of CNN). Xu et al. (2021) applied the DenseNet CNN architecture to segment and quantify intracranial hemorrhage from 3,000 CT imaging datasets. To our knowledge, only one study (Kuang et al., 2019) proposed a semi-automatic method based on D-Unet for hemorrhage segmentation following reperfusion therapies with 30 patients. However, Deep Learning methods require a large amount of expertly labeled data for model training and have high computational demands and so may not be applicable in routine clinical practice. A rapid, accessible and accurate method is needed for segmenting post-treatment hemorrhage for stroke patients in clinical settings.

Computational efforts to date have primarily focused on segmentation of primary ICH, with less attention paid to the characterization of hemorrhage after hyperacute treatment for acute ischemic stroke. One reason relates to the fact that hemorrhage after reperfusion therapies is typically more heterogeneous than primary ICH and therefore challenging to accurately detect and segment. Bleeding after reperfusion therapies can vary in size, location, and pattern, and can be diffuse, scattered, or confined to a specific region. In addition, reperfusion therapy, in particular mechanical thrombectomy, commonly causes contrast extravasation and/or contrast enhancement of infarct on CT performed afterwards. This can be difficult to distinguish from hemorrhage. Partial voluming effects, calcification and movement artifacts can also mimic or obscure the appearance of hemorrhage (Kuang et al., 2019), complicating the detection and segmentation process.

This study aimed to automatically segment hemorrhagic regions on follow-up non-contrast head CT (NCCT) for stroke patients treated with endovascular thrombectomy (EVT). We applied a semi-automatic approach with adaptive thresholding methods based on local pixel intensity. This methodology does not require a large training data set, and so minimizes the computational burden. We hypothesize that this tool would segment HT in a fast and reliable fashion.

## 2 Methods

### 2.1 Patient population and CT imaging

We included ischemic stroke patients from the International Stroke Perfusion Imaging Registry (INSPIRE) at 10 stroke centers between July 2015 and March 2022. Patient inclusion and exclusion criteria were as follows: patients with anterior circulation occlusions; successful reperfusion after EVT [defined as a modified Treatment in Cerebral Ischemia (mTICI) score at the end of EVT of 2b-3]; Type 2 hemorrhagic infarction (HI2) or parenchymal hematoma (PH) within 48 h of EVT [Heidelberg Bleeding Classification (von Kummer et al., 2015)]. Regarding hemorrhage type, only intracerebral hemorrhage was included while intraventricular hemorrhage (IVH), extradural hematoma (EDH), subarachnoid hemorrhage (SAH), and subdural hematoma (SDH) were excluded. Written informed consent was obtained from all participants, and the INSPIRE study was approved by the local ethics committees (Gao et al., 2020).

Follow-up NCCT was used to segment hemorrhagic lesions. All imaging data were acquired using CT scanners from a single manufacturer (SIEMENS Medical Systems). All imaging data were in DICOM (Digital Imaging and Communication in Medicine) format and were anonymized. The slice thickness ranged from 4 to 5 mm and the number of axial slices ranged from 24 to 46. The matrix size ranged from 512 × 512 to 512 × 680 and field of view ranged from 164 mm × 197 mm to 320 mm × 320 mm.

HT types on follow-up NCCT were determined by two stroke neurologists independently. To determine the ground truth for hemorrhage, a binary mask containing bleeding regions of each patient was manually delineated using ITK-SNAP (Yushkevich et al., 2006) software by a trained observer and verified by an experienced stroke neurologist.

### 2.2 Semi-automatic segmentation

**Figure 1:**
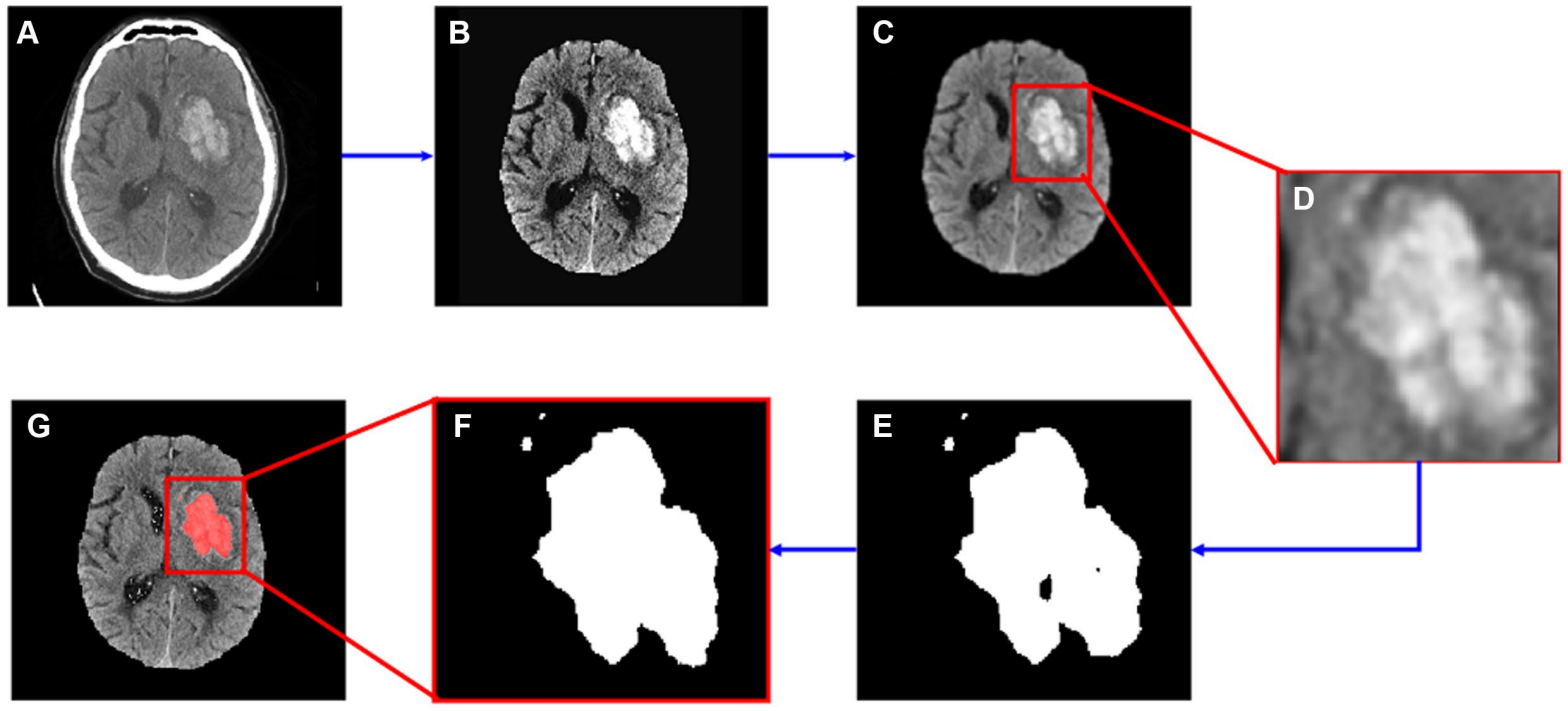
Segmentation flow map. **(A)** The original image; **(B)** image after skull stripping; **(C)** image after noise reduction and edge enhancement; **(D)** cropped image based on manually input ROI data; **(E)** segmented hemorrhage area; **(F)** segmented image after morphological operations; **(G)** segmented result.

All image processing was performed in Python (v 3.10.6) using the SimpleITK toolkit (v2.1.1.2) (Lowekamp et al., 2013; Beare et al., 2018; Yaniv et al., 2018). The proposed algorithm consisted of three steps: pre-processing, adaptive thresholding, and post-processing. The pre-processing step involved skull stripping, noise reduction and edge enhancement. Images were skull stripped using an in-house algorithm based on K-Means clustering and Level Sets methods. Noise reduction was accomplished by applying a Gaussian filter (σ = 1) and edges were enhanced using the Unsharp Masking filter (σ = 1). In the next step, a region of interest (ROI) was manually selected by the user in the two-dimensional (2D) image, then an adaptive thresholding method was applied to segment the bleeding area within the ROI. Further details on adaptive thresholding are in Section 2.2.1. Finally, during post-processing, a binary morphological closing operation (kernel size = 4) was performed to fill holes. The entire process is illustrated in Figure 1.

#### 2.2.1 Adaptive thresholding

Using a fixed thresholding method for all patients was not suitable due to the heterogeneity of density across patient images. Therefore, we adopted an adaptive method to customize each patient’s threshold based on local voxel intensity characteristics. As a first step, the segmentation threshold for each patient was set to 50–80 Hounsfield Units (HU), and an initial segmentation mask was generated for each patient. Through visual inspection, cases were categorized as “under-segmented,” “well-segmented,” or “over-segmented” (Classification column in Table 1). Then, we calculated the intensity mean and standard deviation (SD) of the segmented area. By analyzing the SD values of each case, we found that lower SD values (narrower band of intensity values) were consistent with under-segmentation, and higher SD values (larger band of intensity values) consistent with over-segmentation. Specifically, cases with SD > 11 were consistently over-segmented, those with SD < 6 were mostly under-segmented, and SD values between 6 and 11 indicated well-segmented cases. To address under- and over-segmentation, thresholds were adjusted accordingly: from 50–80 to 45–75 for under-segmented cases, and from 50–80 to 55–85 for over-segmented cases. Further refinement was conducted based on SD values: thresholds were adjusted to 40–70 for cases with SD between 0 and 2.3, and to 35–65 for cases with SD equal to 0. Subsequent iterations were performed to assess the effectiveness of these adjustments. Based on the experimental results, the segmentation rules with updated thresholds were determined empirically and the rules are shown in Table 1.

**Table 1:** Adaptive thresholding rules.

SD value	Classification	Updated threshold
SD = 0	Under-segmented	35–65
0 < SD ≤ 2.3	Under-segmented	40–70
2.3 < SD ≤ 6	Under-segmented	45–75
6 < SD ≤ 11	Well-segmented	50–80
SD > 11	Over-segmented	55–85

### 2.3 Performance evaluation

The Dice Similarity Coefficient (DSC) (Zou et al., 2004), the area under the receiver operating characteristic curve (AUC), Sensitivity, Specificity and Lin’s concordance correlation coefficient (Lin’s CCC) (Lawrence and Lin, 1989) were used to evaluate performance. The DSC measures the spatial overlap between the manually segmented region (the ground-truth) and the automatic segmented region. It ranges from 0 (no overlap) to 1 (perfect overlap) and is commonly used to evaluate segmentation performance. Specifically, DSC can be classified into the following six categories (Landis and Koch, 1977; Pajula et al., 2012; Werdiger et al., 2023): 0, “No agreement,” 0–0.2, “Slight agreement”; 0.2–0.4, “Fair agreement”; 0.4–0.6, “Moderate agreement”; 0.6–0.8; “Substantial agreement”; “0.8–1”; “Almost perfect agreement.” Generally, an AUC ≥ 0.8 is considered acceptable (Nahm, 2022). Sensitivity was defined as the number of true positives divided by the total number of patient cases that belong to the positive cluster; specificity is defined as the number of true negatives divided by the total number of patient cases that belong to the negative cluster (Weng and Poon, 2008). In the context of imaging segmentation, sensitivity is an attribute that measures how well the algorithm identifies the regions of interest. This means a lower sensitivity indicates the case is under-segmented. Specificity measures how well the algorithm identifies the background, meaning a lower specificity indicates the case is over-segmented. Lin’s CCC coefficient describes the agreement between two different measurements of the same variable. A higher Lin’s CCC indicates greater concordance, with values near 1 indicating perfect concordance, values near −1 indicating perfect discordance, and values near 0 indicating no concordance. Statistical analyses were conducted with Stata software package (V.13.1; Stata, College Station, Texas, United States).

Besides, a state-of-the-art segmentation model SAMIHS (Wang et al., 2023) was included in the performance comparison to evaluate the effectiveness of the proposed model. SAMIHS was performed on a Linux workstation with 2.9 GHz Xeon processors (Intel, Santa Clara, CA, United States) with 32 GB memory and a NVIDIA Tesla V100 GPU. It was based on Segment Anything Model (SAM)’s pre-trained ViT-B variant and fine-tuned in 200 epochs with a batch size of 2. A slice-level 5-fold cross-validation were applied for all the experiments. The training process took approximately 10.5 h in each cross-validation iteration.

## 3 Results

In total, 51 patients from 10 hospitals across Australia and China were enrolled of which 28 had HI2 and 23 had PH. The flow diagram of patient inclusion and exclusion is shown in Supplementary Figure S1. The mean age of enrolled patients was 73.6 (SD = 11). Among these patients, 54.9% (28 out of 51) were male. Across all cases, the mean volume of the hemorrhage region was 11.53 mL (SD = 12.06). The mean volume for PH cases was 17.28 mL (SD = 12.44, min-max = 3.89–53.44) and for HI2 cases, 6.81 mL (SD = 9.59, min-max = 0.08–35.40).

The auto-segmentation model was built using PyCharm software (JetBrains s.r.o.) on a PC with CPU, 11th Gen Intel(R) Core (TM) i5-1145G7 @ 2.60GHz and 16 GB memory. The mean processing time for each patient case was 2.7 s (SD 0.7).

Across all cases, the mean DSC was 0.66 (SD = 0.17, min-max = 0.23–0.92). The DSC for PH patients was 0.73 (SD = 0.14, min-max = 0.23–0.92), higher on average than HI2 patients, who had a mean DSC of 0.61 (SD = 0.18, min-max = 0.23–0.88). Additional results are shown in Table 2.

**Table 2:** Results.

	Number of cases	Mean DSC (SD)	Mean AUC (SD)	Mean sensitivity (SD)	Mean specificity (SD)
Total	51	0.66 (0.17)	0.87 (0.08)	0.81 (0.17)	0.93 (0.08)
HI2	28	0.61 (0.18)	0.87 (0.07)	0.84 (0.14)	0.90 (0.10)
PH	23	0.73 (0.14)	0.86 (0.09)	0.77 (0.19)	0.96 (0.04)

Dice similarity coefficient (DSC), Area under curve (AUC), Sensitivity and Specificity are shown for all cases and for subtypes.

**Figure 2:**
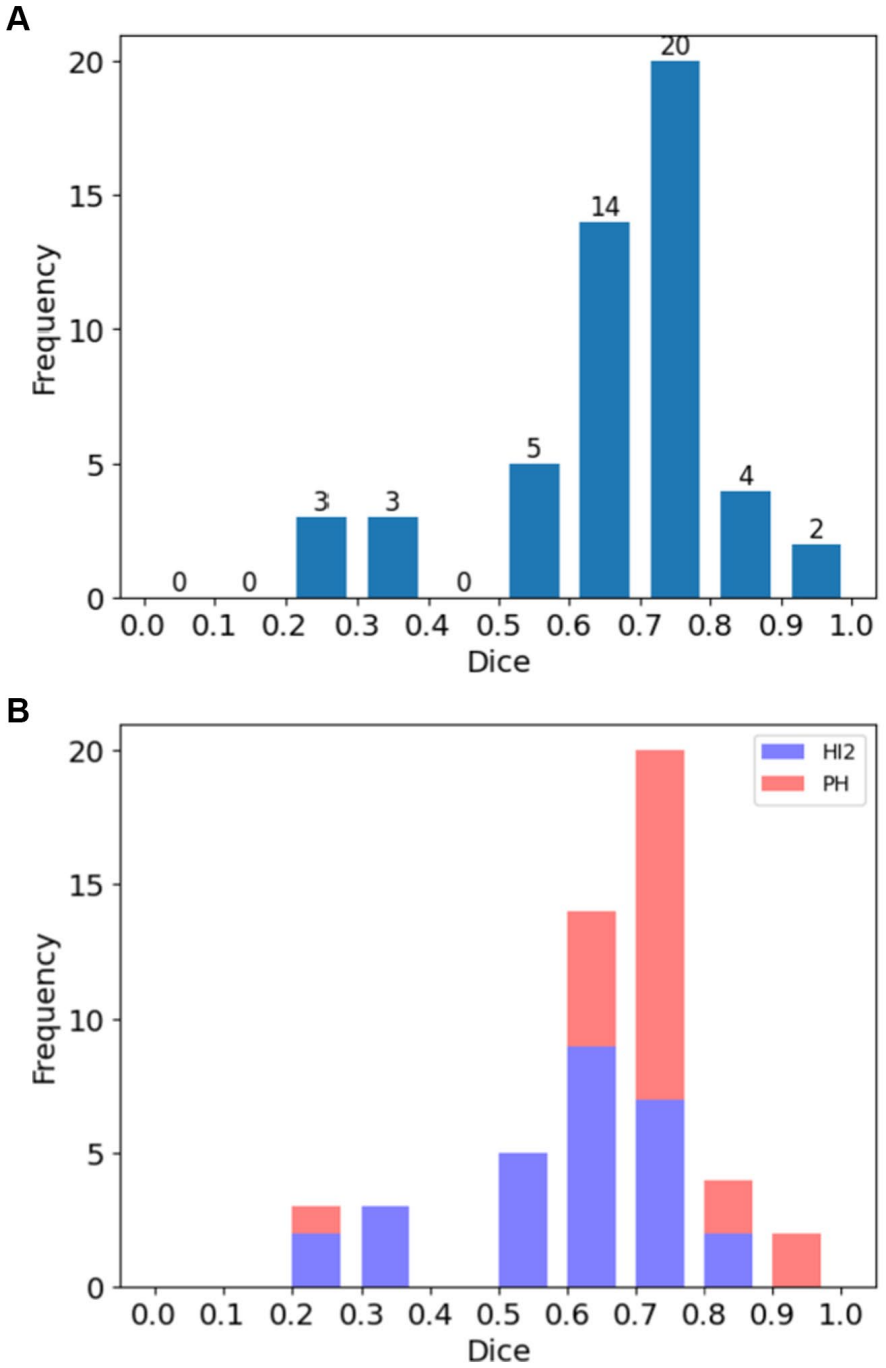
Histograms of Dice Similarity Coefficients (DSC) for patients in the study. **(A)** Distribution of DSC across all cases, and **(B)** the same showing the distribution according to hemorrhage type (HI2: Type 2 hemorrhagic infarction; PH: parenchymal hematoma).

Figure 2 demonstrates the distribution of DSC for all cases (Figure 2A) and for each HT type (Figure 2B). Overall, 78.4% (40 out of 51) cases achieved substantial agreement or more (DSC > 0.6). On the contrary, 11.8% (6 out of 51) cases had fair agreement (DSC between 0.2–0.4). Among these cases, three had very small bleeding volumes, ranging from 0.08 mL to 0.48 mL. The case with the lowest Dice (0.23) had very blurry boundaries and low contrasts to the background, making it difficult to distinguish even for expertise. As a result, the model classified it as under-segmented according to the rules of Table 1 although it was over-segmented based on visual inspection.

**Figure 3:**
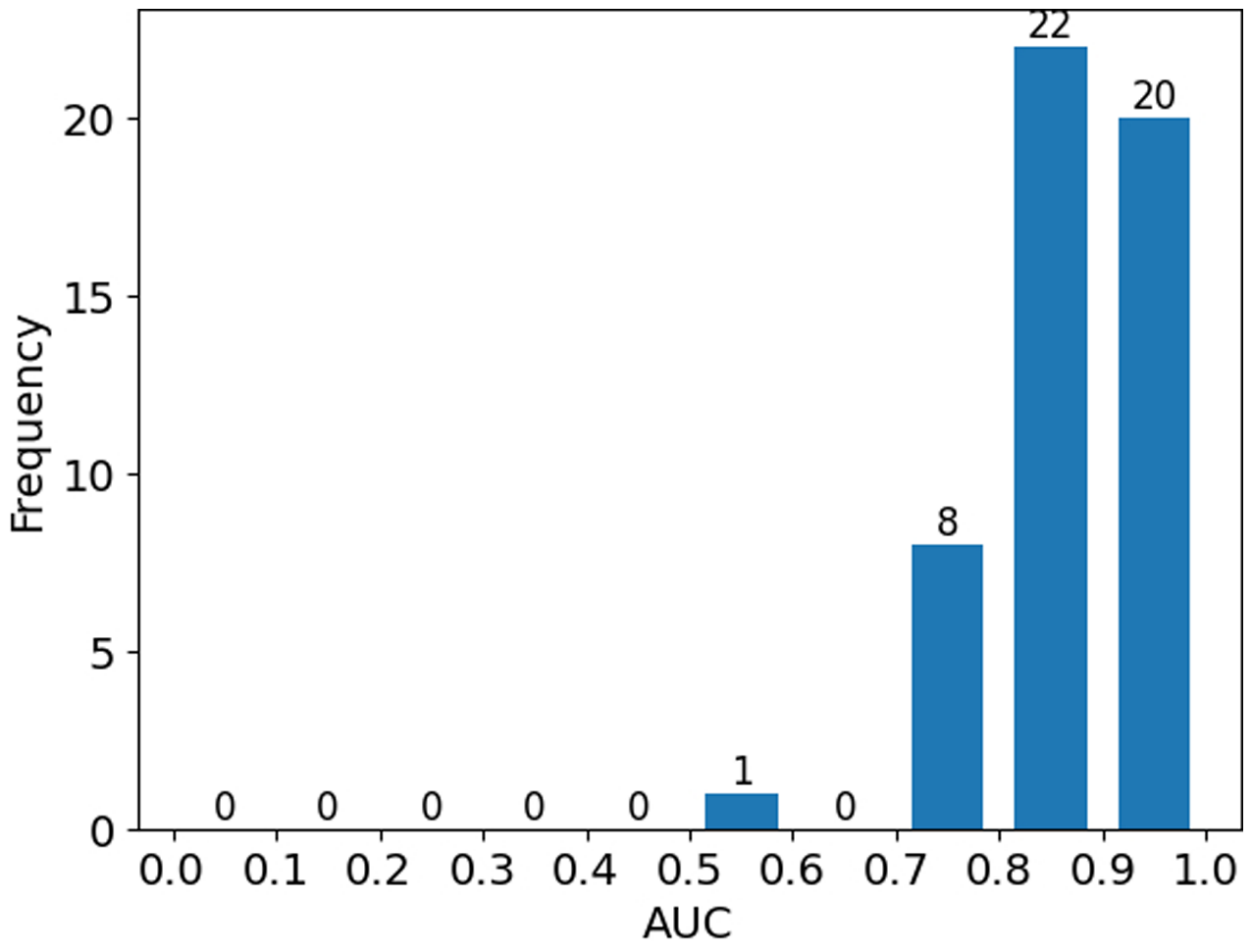
Histogram showing the distribution of ROC-AUC scores across all patients in the study.

Figure 3 illustrates the distribution of AUC score; most cases (98.0%, 50 out of 51) achieved AUC above 0.7 and only one cases had AUC below 0.6.

**Figure 4:**
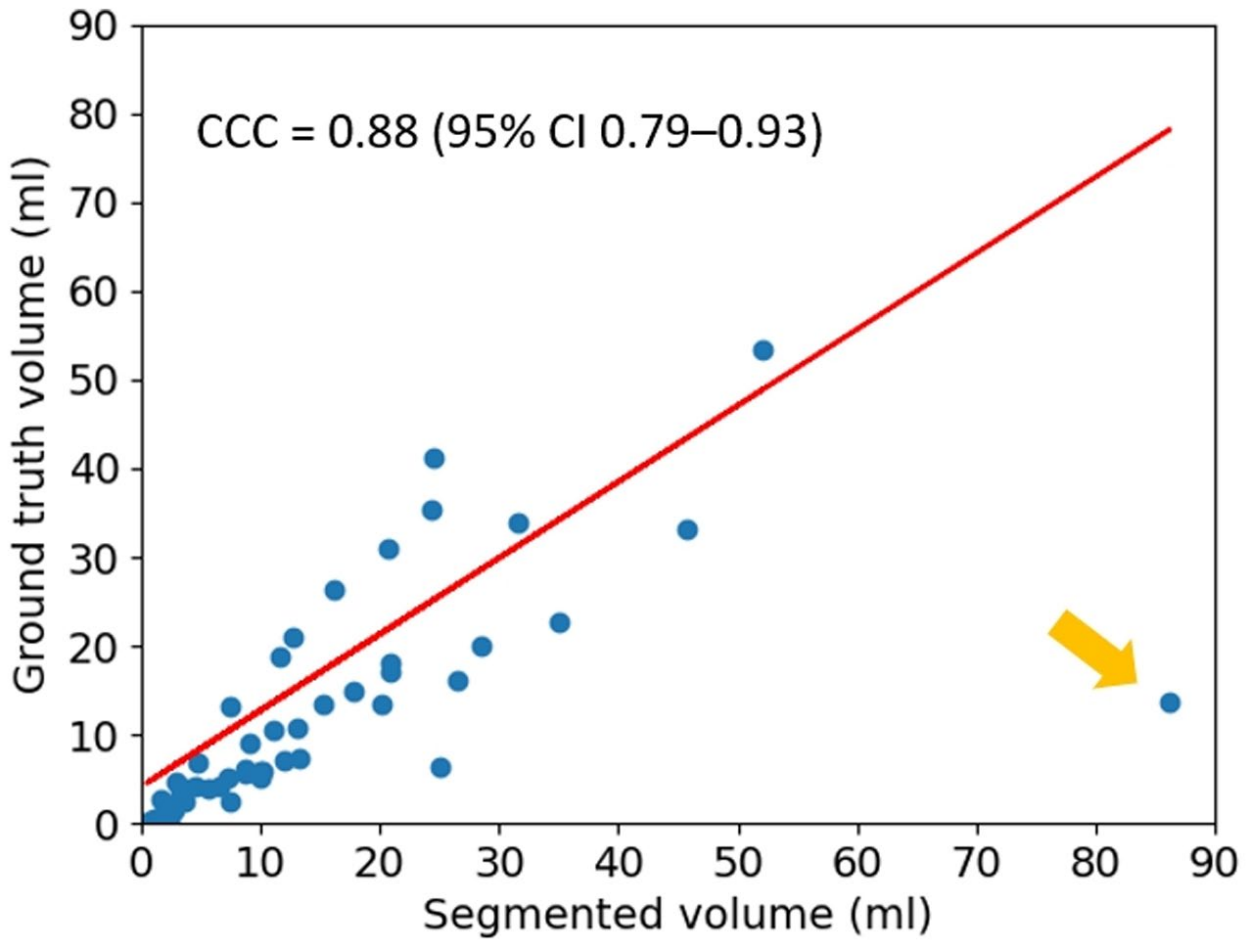
Lin’s Concordance Correlation Coefficient (CCC). The result for Lin’s CCC is 0.88 (95% CI 0.79–0.93). The yellow arrow indicates a single outlier.

The segmented results were evaluated against the ground truth using Lin’s CCC and the result is shown in Figure 4. The coefficient was 0.88 (95% CI 0.79–0.93), indicating a strong agreement between the segmented results and the ground truth. Yellow arrow indicates a single outlier in Figure 4, which corresponds to the mis-classified case mentioned above.

**Figure 5:**
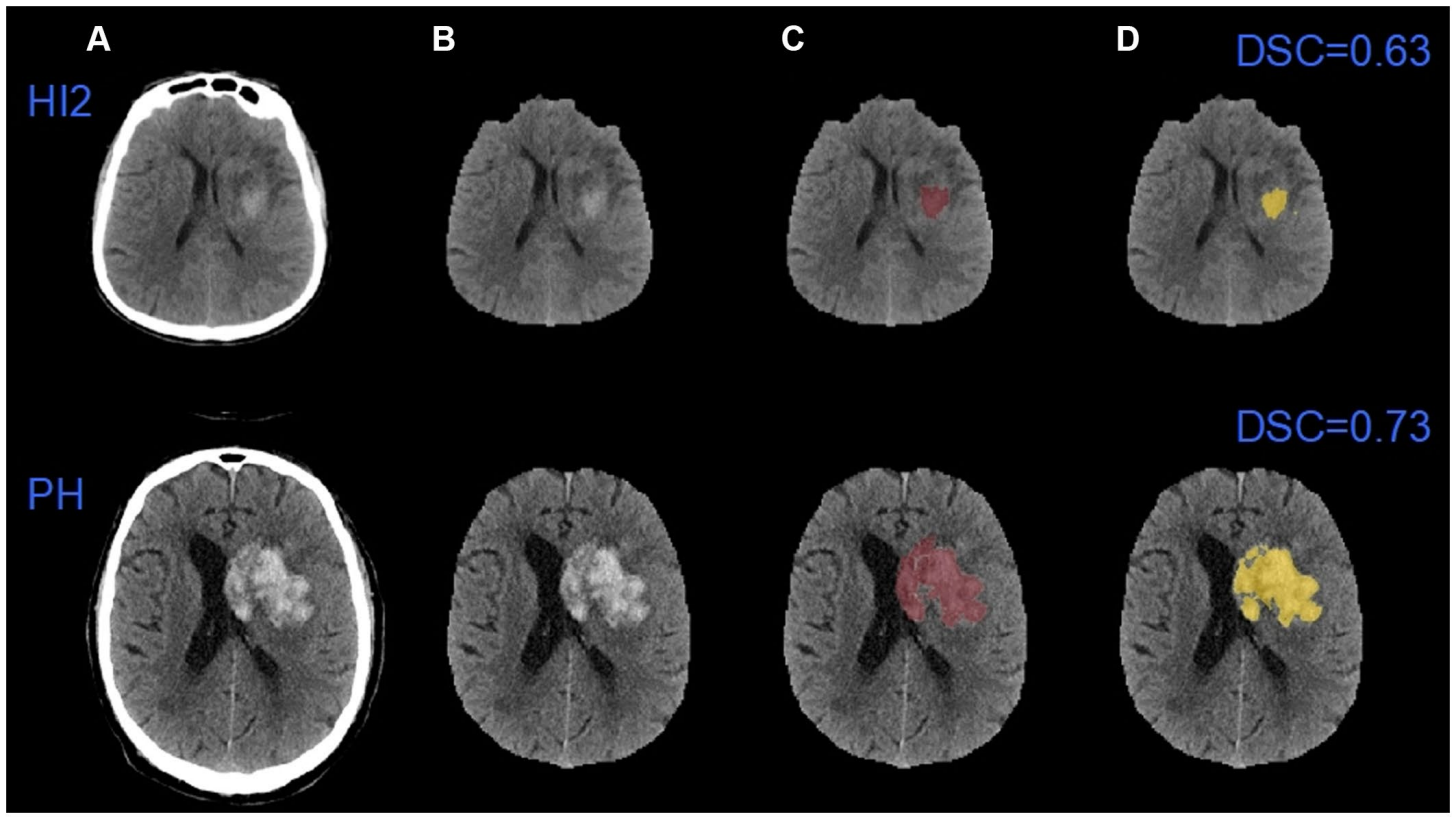
An example of automatic segmentation results. **(A)** The original images; **(B)** images after removing the skulls; **(C)** the ground truth images; **(D)** the auto-segmented images. HI2: Type 2 hemorrhagic infarction; PH: parenchymal hematoma.

Figure 5 shows automated segmentations of one HI2 case and one PH case, respectively. The DSC for the HI2 case was 0.63 and the DSC for the PH case was 0.73.

**Figure 6:**
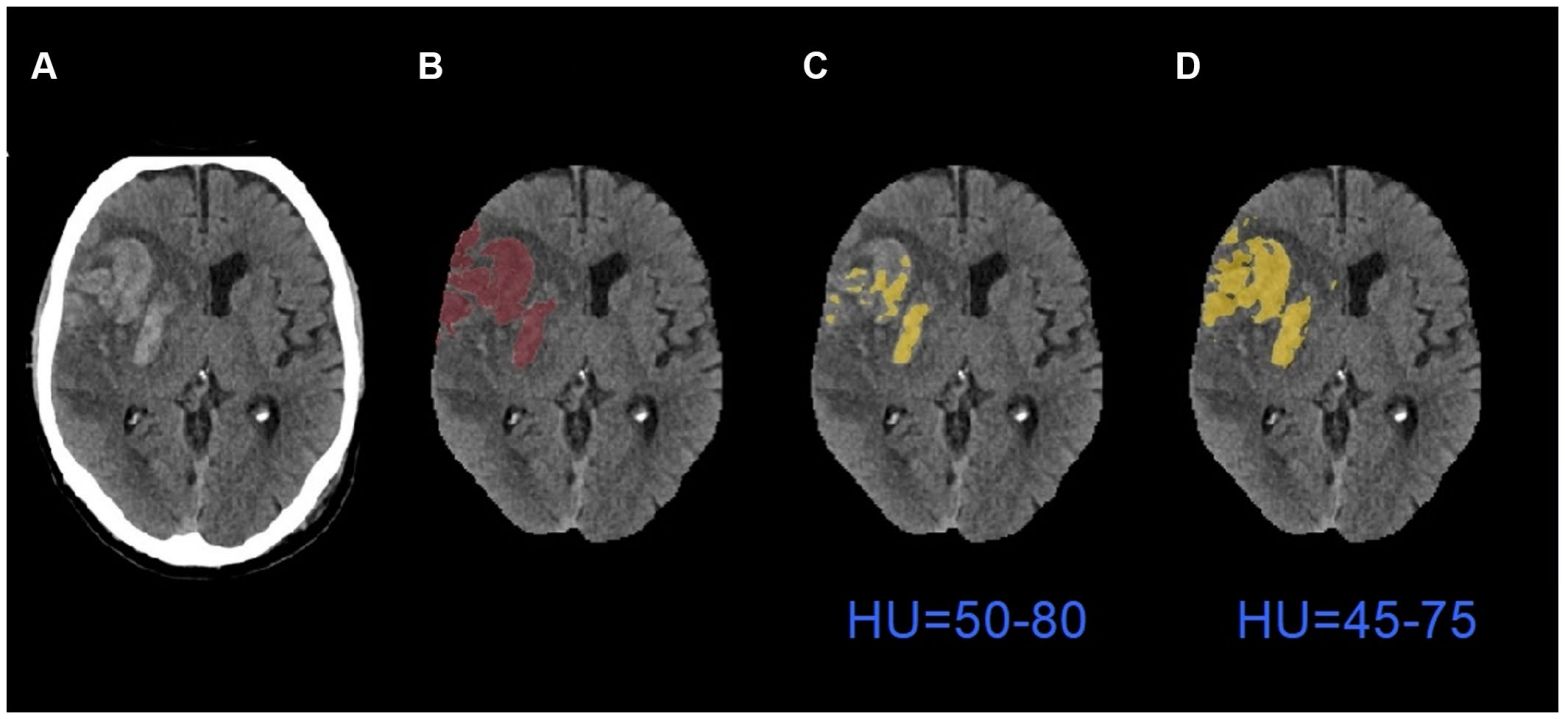
An example of segmentation results before and after applying adaptive thresholding. **(A)** The original images; **(B)** the ground truth images; **(C)** the segmentation results with global thresholding method; **(D)** the segmentation results with adaptive thresholding method. HU: Hounsfield Units.

Table 3 shows the comparison of performance between default global thresholding method and the proposed adaptive thresholding method. The proposed adaptive thresholding method achieved higher DSCs than the default global thresholding method for both HI2 and PH cases. Figure 6 illustrates an example that improved performance after applying the adaptive thresholding method. It is clear from visual inspection that the default global thresholding method failed to capture the whole hemorrhage area and the result was heavily under-segmented. After applying the adaptive thresholding method, the segmentation results agreed much better with the ground truth.

**Table 3:** Segmentation performance before and after applying adaptive thresholding method.

	Mean DSC (SD)		
	Total	HI2	PH
Global thresholding	0.57 (0.25)	0.49 (0.26)	0.66 (0.20)
Adaptive thresholding	0.66 (0.17)	0.61 (0.18)	0.73 (0.14)

Table 4 shows the comparison of performance between the proposed adaptive thresholding method and the SAMIHS model. Our method exhibited comparable performance to SAMIHS, which leverages advanced image segmentation techniques, demonstrating the effectiveness of our approach. Notably, the average training time for SAMIHS was 12.3 min per patient, significantly exceeding the average processing time of our method (2.7 s per patient). Further information on the SAMIHS training results for 5-fold cross-validation is presented in Supplementary Table S1.

**Table 4:** Performance comparison between the adaptive thresholding method and a state-of-art method.

	Mean DSC (SD)		
	Total	HI2	PH
Adaptive thresholding	0.66 (0.17)	0.61 (0.18)	0.73 (0.14)
SAMIHS (Wang et al., 2023)	0.66 (0.02)	0.64 (0.07)	0.69 (0.03)

## 4 Discussion

We developed a semi-automated method for the segmentation of hemorrhagic transformation after endovascular thrombectomy for patients with acute ischemic stroke. Our results, based on a multicenter dataset, show that this method was able to extract hemorrhage from follow-up NCCT images with high accuracy, and, importantly, with very low computational requirements. To our knowledge, this is the first study to perform automated lesion segmentation of post-treatment hemorrhage for acute stroke patients that groups the results based on the radiological severity of HT.

The workflow used three main steps to improve the performance of the algorithm. First, we applied image preprocessing methods to remove skull and artifact, as these could confuse the segmentation algorithm. Second, coordinates of the region of interest around the hemorrhage area needed to be provided to restrict the segmentation. Although information on the region of interest requires manual input, identifying the region of interest is relatively straightforward and does not require expertise. Specifying the region of interest can help to exclude unrelated regions such as those caused by background noise and artifact, enhancing segmentation performance (Kushibar et al., 2018; Abramova et al., 2021). Third, we applied adaptive thresholding techniques that considered local pixel intensity properties. Due to the substantial intensity variability of hemorrhage areas across different patients, a significant increase in performance using customized thresholding rather than global thresholding was expected, as has been shown in previous work (Zhang et al., 2013). In our study, the proposed algorithm automatically decreased the threshold for cases labeled as under-segmented and increased the threshold for cases labeled as over-segmented, achieving a satisfactory result.

The proposed method achieved a mean DSC of 0.66 with 51 patients, which, considering HT is more difficult to discriminate than primary ICH, is higher than some past studies that only segmented primary ICH, with scores of 0.28 and 0.55 reported, respectively, in Hssayeni et al.’s (Hssayeni et al., 2020) and Boers et al.’s studies (Boers et al., 2014). The result was comparable to the scores of 0.653 reported in Kuang et al.’s study of HT with 30 patients (Kuang et al., 2019) and 0.697 reported in Yao et al.’s study of primary ICH with a dataset of 120 CT scans (Yao et al., 2020), albeit lower than the scores of 0.862 with 76 cases reported in Abramova et al.’s study (Abramova et al., 2021), and 0.90 with 1,000 images reported by Xu et al. (2021). In addition, the DSC for HI2 cases in our study was 0.61 with 28 patients, compared to less than 0.45 for small primary hemorrhage region segmentation with 190 patients reported in Li et al.’s study (Li et al., 2020). The DSC for PH cases was 0.73 with 23 patients, compared to 0.803 for large hemorrhage region segmentation with 190 patients reported in the same study.

Segmentation performance for PH cases was better than for HI2 cases (a mean DSC of 0.73 for PH cases versus a mean DSC of 0.61 for HI2 cases, as shown in Table 2). This is not surprising. In comparison to PH, HI2 cases have smaller hemorrhage volumes, lower contrast-to-background definition, and more varied shapes, making them difficult to segment even by experienced neurologists. A similar pattern has also been found in previous work (Li et al., 2020; Wang et al., 2023). In any case, better performance for PH cases makes our results more clinically relevant compared to the SAMIHS model (Wang et al., 2023) (DSC of 0.73 versus DSC of 0.69). This is because compared to HI2, PH is more likely to predict a poor prognosis (Yaghi et al., 2014; Sun et al., 2023). Accurate segmentation of PH could therefore facilitate early therapeutic, prognostic, and rehabilitation decisions.

When compared with deep learning-based methods (Kuang et al., 2019; Yao et al., 2020; Abramova et al., 2021; Xu et al., 2021), our study has some key advantages. First, the processing time of our method is significantly faster. The mean processing time for each patient is 2.7 s in our study, which compares favorably with Abramova et al.’s (2021) reported mean processing time of 17 s and Kuang et al.’s (2019) reported mean processing time of 4.5 min. Our method is also faster compared to other non-Deep Learning-based methods (Pszczolkowski et al., 2019). In addition, the proposed method does not require a large dataset for training or high computational power and may be run on a standard CPU. Furthermore, the proposed method is user friendly for a clinical setting and user input is not a barrier to implementation. Another advantage of our tool in clinical settings is the utilization of follow-up NCCT to establish the ground truth for hemorrhage. While contrasted CT or Magnetic Resonance Imaging (MRI) scans may provide better options in determining permanent brain lesions, NCCT remains the principal modality to diagnose post-treatment hemorrhage due to its wide availability, low cost, and fast image acquisition (Siddiqui et al., 2011; Heit et al., 2017). This characteristic facilitates the seamless integration of our method across a broader spectrum of clinical scenarios. Therefore, while Deep Learning methods may report higher performance metrics, our method is almost as accurate and more practical.

While Abramova et al. (2021) and Xu et al. (2021) achieved higher accuracy than our method, it is prudent to note when interpreting their performance metrics that their imaging data was collected from a single center, whereas our study dataset is drawn from multiple sites (and countries). If an algorithm is developed and tested on data from a single site, it often fails to generalize (and achieve the same accuracy) when tested under different conditions (Torpmann-Hagen et al., 2022). Furthermore, the hemorrhage volumes in Xu et al.’s study (Xu et al., 2021) were considerably larger (up to 180 mL versus up to 53.44 mL in our study), which may account for the higher DSC values due to the well-documented trend of the DSC favoring larger segmentation volumes (Chang et al., 2018; Ironside et al., 2019). To our knowledge only one previous report used Deep Learning-based methods to identify and segment HI2 (Li et al., 2020), and this study had a lower performance than ours (DSC < 0.45 versus DSC of 0.61).

There are several caveats to our study. First, our method is semi-automated and not fully automated. Manual entry of the ROI data is required for each case. Semi-automated methods are necessary steps toward full automation and a fully automated method will be developed in a follow-up study. Second, our adaptive thresholding method relies on hard cut-offs under the assumption that the SD indicates whether a case is over- or -under segmented. However, it is not always the case. Although most cases were successfully adapted, one case was misclassified, resulting in poor agreement with the ground truth. The image in this case had indistinct boundaries between hemorrhage and normal brain tissue, making it particularly difficult to segment even for human experts. This effect may be mitigated by introducing an additional parameter based on image characteristics to classify cases in a more robust fashion. Further development is necessary. Third, the sample size is relatively small, a further validation with external database is warranted to confirm the results. Although the imaging data was collected from multiple centers, it used CT scanners from a single manufacturer. Hard cut-offs may need to be adjusted when applying the method to a different dataset. Fourth, this tool is designed to segment the hemorrhage after reperfusion therapy, it would be more meaningful in clinical settings if the tool can predict hemorrhagic regions using baseline CT scans.

## 5 Conclusion

We propose a semi-automated approach to the segmentation of post-reperfusion treatment hemorrhage on NCCT for patients with acute ischemic stroke. The algorithm demonstrated excellent accuracy and strong correlation with the gold standard of manual segmentation by expert human raters. This rapid tool has the potential to assist with predicting prognosis following hemorrhagic transformation in stroke patients who undergo endovascular thrombectomy.

## Data availability statement

The original contributions presented in the study are included in the article/Supplementary material, further inquiries can be directed to the corresponding authors.

## Ethics statement

The studies involving humans were approved by The Hunter New England Area Health Service Human Research Ethics Committee. The studies were conducted in accordance with the local legislation and institutional requirements. Written informed consent for participation was not required from the participants or the participants’ legal guardians/next of kin in accordance with the national legislation and institutional requirements.

## Author contributions

JS: Writing – review & editing, Writing – original draft, Software, Investigation, Formal analysis, Data curation. FW: Writing – review & editing, Supervision, Software, Resources, Methodology. CB: Writing – review & editing, Validation. CC: Writing – review & editing, Validation, Resources. QY: Writing – review & editing, Supervision. AB: Writing – review & editing, Supervision, Methodology, Conceptualization. LL: Writing – review & editing, Supervision, Project administration, Methodology. MP: Writing – review & editing, Supervision, Methodology, Conceptualization.

## Supplementary material

Supplementary Figure S1 Patient selection flow diagram.
