# Supplementary figures and images for: Automatic segmentation of hemorrhagic transformation on follow-up non-contrast CT after acute ischemic stroke
Source: Front Neuroinform. 2024 Apr 16;18:1382630. doi: 10.3389/fninf.2024.1382630 (PMC11058994; doi:10.3389/fninf.2024.1382630)

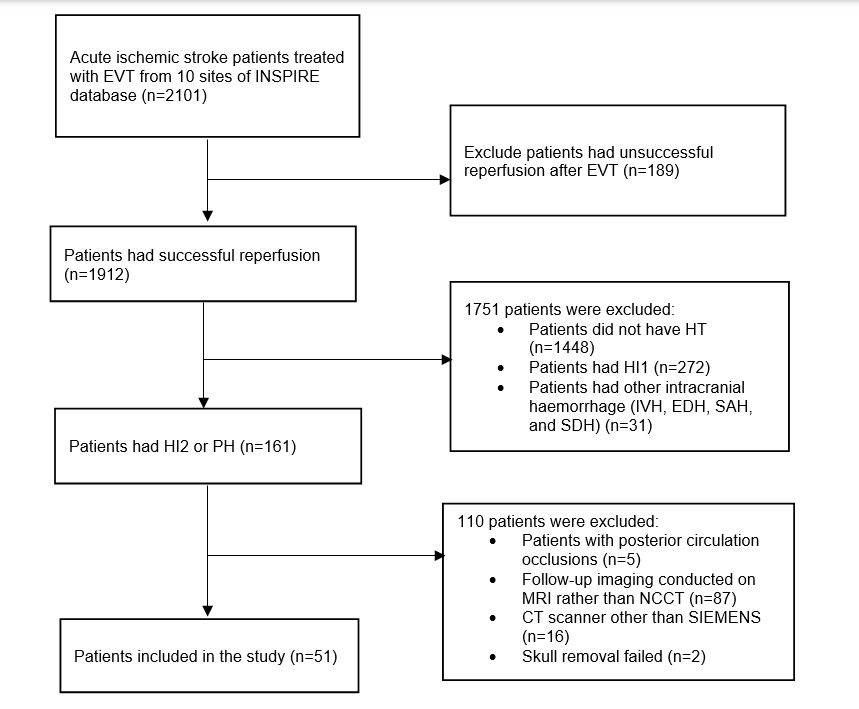

Supplement: Supplementary Figure S1 — Patient selection flow diagram. [file Image_1.JPEG]
